# Theoretical prediction of electronic properties and contact barriers in a metal/semiconductor NbS_2_/Janus MoSSe van der Waals heterostructure†

**DOI:** 10.1039/d3na00852e

**Published:** 2024-01-09

**Authors:** P. H. Nha, Chuong V. Nguyen, Nguyen N. Hieu, Huynh V. Phuc, Cuong Q. Nguyen

**Affiliations:** a Faculty of Electrical Engineering, Hanoi University of Industry Hanoi 100000 Vietnam nhaph@haui.edu.vn; b Department of Materials Science and Engineering, Le Quy Don Technical University Hanoi Vietnam chuong.vnguyen@lqdtu.edu.vn; c Institute of Research and Development, Duy Tan University Da Nang 550000 Vietnam nguyenquangcuong3@duytan.edu.vn; d Faculty of Natural Sciences, Duy Tan University Da Nang 550000 Vietnam; e Division of Theoretical Physics, Dong Thap University Cao Lanh 870000 Vietnam hvphuc@dthu.edu.vn

## Abstract

The emergence of van der Waals (vdW) heterostructures, which consist of vertically stacked two-dimensional (2D) materials held together by weak vdW interactions, has introduced an innovative avenue for tailoring nanoelectronic devices. In this study, we have theoretically designed a metal/semiconductor heterostructure composed of NbS_2_ and Janus MoSSe, and conducted a thorough investigation of its electronic properties and the formation of contact barriers through first-principles calculations. The effects of stacking configurations and the influence of external electric fields in enhancing the tunability of the NbS_2_/Janus MoSSe heterostructure are also explored. Our findings demonstrate that the NbS_2_/MoSSe heterostructure is not only structurally and thermally stable but also exfoliable, making it a promising candidate for experimental realization. In its ground state, this heterostructure exhibits p-type Schottky contacts characterized by small Schottky barriers and low tunneling barrier resistance, showing its considerable potential for utilization in electronic devices. Additionally, our findings reveal that the electronic properties, contact barriers and contact types of the NbS_2_/MoSSe heterostructure can be tuned by applying electric fields. A negative electric field leads to a conversion from a p-type Schottky contact to an n-type Schottky contact, whereas a positive electric field gives rise to a transformation from a Schottky into an ohmic contact. These insights offer valuable theoretical guidance for the practical utilization of the NbS_2_/MoSSe heterostructure in the development of next-generation electronic and optoelectronic devices.

## Abstract 2

The emergence of van der Waals (vdW) heterostructures, which consist of vertically stacked two-dimensional (2D) materials held together by weak vdW interactions, has introduced an innovative avenue for tailoring nanoelectronic devices.

## Graphical abstract

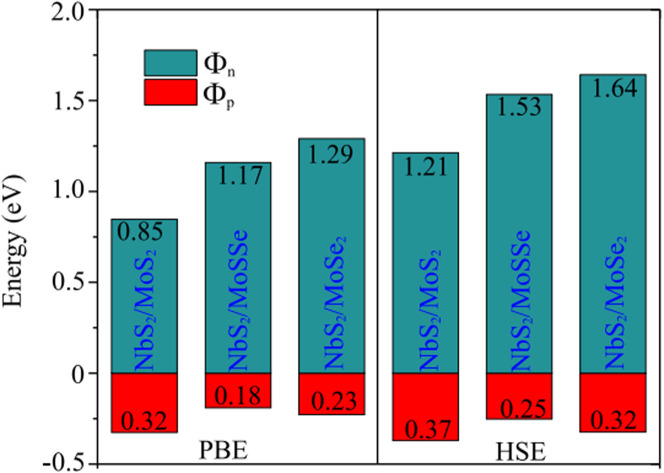

## 1 Introduction

In recent years, significant attention has been directed towards two-dimensional (2D) materials due to their remarkable attributes and diverse potential applications.^1–3^ The discovery of graphene^4^ nearly two decades ago marked the inception of a transformative era in materials science and nanotechnology, fueling extensive investigations into the distinctive characteristics and promising uses of 2D materials. Over this period, researchers have identified and synthesized numerous 2D materials, each possessing unique properties and potential applications, including transition metal dichalcogenides (TMDs),^5,6^ MXenes^7–10^ and the MA_2_Z_4_ family (M = Mo, W; A = Si, Ge; Z = N, P).^11–13^ As research on 2D materials continues to advance, it becomes increasingly evident that their versatility and intriguing properties have the potential to reshape various fields, presenting both exciting challenges and opportunities for innovative technologies.^14–17^ Among the myriad of 2D materials, the family of 2D TMDs has emerged as a particularly captivating and extensively studied category due to its unique attributes and broad range of applications, including electronics,^18^ optoelectronics,^19^ energy storage^20^ and photocatalysis.^6^ 2D TMDs can exhibit either metallic or semiconductor behavior. For example, monolayers of MoS_2_ and MoSe_2_ are known as semiconductors, while NbS_2_ and NbSe_2_ monolayers exhibit metallic characteristics. These 2D materials have been successfully synthesized through various methods such as mechanical exfoliation^21^ or chemical vapor deposition (CVD).^22,23^

Recently, a new category of 2D materials has emerged, namely Janus structures.^24^ Specifically, Janus MoSSe has been successfully synthesized using the chemical vapor deposition (CVD) method.^25,26^ Janus MoSSe can be obtained by either replacing the top-layer sulfur (S) atoms in a MoS_2_ monolayer with selenium (Se) atoms^25^ or by sulfurization of the top layer of selenium atoms in a MoSe_2_ monolayer.^26^ The creation of Janus MoSSe monolayers from MoS_2_ and MoSe_2_ monolayers disrupts their out-of-plane mirror symmetry, resulting in the emergence of unique properties that distinguish them from traditional TMD monolayers, such as Rashba splitting and strong out-of-plane piezoelectricity.^27,28^ Interestingly, the electronic and transport properties of a Janus MoSSe monolayer can be controlled by using the layer thickness,^29^ vacancies^30,31^ and strain engineering.^32^ Hence, Janus MoSSe monolayers hold promise for diverse applications, including gas sensing,^33^ water splitting^34,35^ and Li-ion batteries.^36^

Notably, the development of van der Waals (vdW) heterostructures, composed of distinct 2D materials vertically stacked and held together by weak vdW interactions, has introduced a novel approach to tailor nanoelectronic devices.^37,38^ This stacking arrangement allows for the preservation of the intrinsic properties of the constituent materials to a significant extent. Furthermore, the combination of different 2D materials often leads to unexpected and novel behaviors, opening up exciting possibilities in nanoelectronics. In recent times, the majority of investigations have centered on pairing two distinct 2D semiconductors. However, the creation of metal/semiconductor heterostructures through the combination of 2D metals and 2D semiconductors plays a pivotal role in advancing electronic device development.^39–41^ Currently, there is growing interest in the combination of metallic NbS_2_ with other 2D semiconductors, as they can form metal/semiconductor heterostructures with either low Schottky barriers or ohmic contacts, depending on the stacking orientations. Hence, in this study, we construct a novel heterostructure by vertically stacking metallic NbS_2_ and Janus MoSSe monolayers and investigate the electronic properties and interfacial characteristics using first-principles predictions. The stacking effects and effects of external electric fields are also explored to enhance the tunability of these heterostructures. We find that the NbS_2_/MoSSe heterostructure exhibits p-type Schottky contacts with small Schottky barriers and low tunneling barrier resistance, highlighting its significant potential for use in electronic devices. Furthermore, we demonstrate that the electronic properties and contact characteristics of the NbS_2_/MoSSe heterostructure can be finely tuned through the application of electric fields. These insights provide valuable theoretical guidance for the practical application of the NbS_2_/MoSSe heterostructure in the development of next-generation electronic and optoelectronic devices.

## 2 Computational model and methods

In this study, we conducted first-principles calculations using the Vienna *ab initio* simulation package (VASP)^42,43^ to investigate the geometric optimization and electronic properties of all considered materials, including NbS_2_, Janus MoSSe monolayers and their combined heterostructure. All calculations were performed within the framework of the generalized gradient approximation (GGA)^44^ for the exchange-correlation energy, utilizing the Perdew–Burke–Ernzerhof (PBE) functional.^45^ To describe the electron–ion interactions, we employed projector augmented wave (PAW) pseudopotentials.^46^ The energy cut-off was set to 510 eV, and we employed a Monkhorst–Pack *k*-point mesh of 9 × 9 × 1. During the structural optimization process, all atomic structures were relaxed until the convergence criteria for force and energy reached 0.01 eV Å^−1^ and 10^−6^ eV, respectively. To eliminate spurious interactions between adjacent layers, a vacuum thickness of 25 Å was introduced along the *z*-direction. We accounted for the weak van der Waals (vdW) interactions inherent in layered structures using DFT-D3 and DFT-D2 methods, which were proposed by Grimme.^47,48^ Furthermore, to rectify the underestimation of the band gap associated with the PBE functional, we employed the Heyd–Sculeria–Ernzerhof (HSE06) functional.^49,50^ Dipole corrections were also incorporated into all calculations. The spin–orbit coupling (SOC) effect has also been added to the calculations.

## 3 Results and discussion

**Fig. 1:**
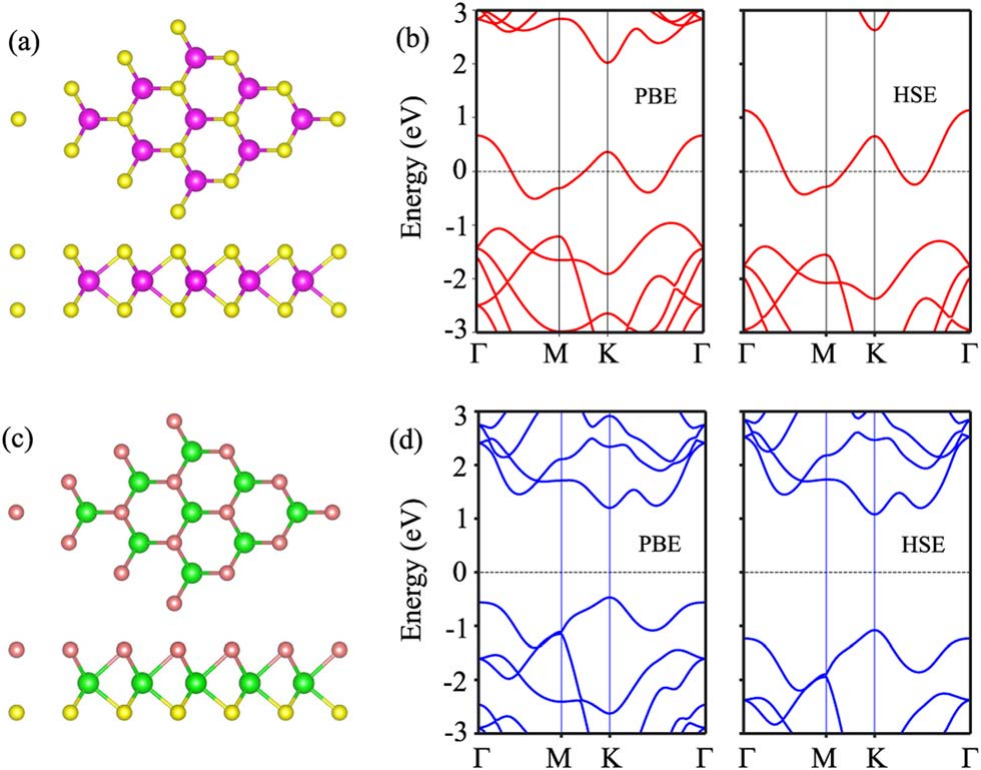
Atomic structures of (a) NbS_2_ and (c) Janus MoSSe monolayers. Yellow, purple, green and brown balls represent the S, Nb, Mo and Se atoms, respectively. Band structures of (b) NbS_2_ and (d) Janus MoSSe monolayers given by using PBE and HSE functionals.

We first examine the atomic and electronic properties of the perfect NbS_2_ and Janus MoSSe monolayers, as depicted in Fig. 1. Both the Nb_2_ and Janus MoSSe monolayers show the same hexagonal crystal structure. The lattice constants of NbS_2_ and Janus MoSSe monolayers are calculated to be 3.32 and 3.28 Å, respectively. These values are in good agreement with the experimental measurements,^25,26,51^ confirming the reliability of our calculations. The electronic band structures of NbS_2_ and Janus MoSSe monolayers are illustrated in Fig. 1(b and d). The NbS_2_ monolayer exhibits metallic behavior, featuring a band that crosses the Fermi level, while Janus MoSSe is a semiconductor with an indirect band gap of 1.67/2.15 eV obtained by using the PBE/HSE functional. Both the maxima of the valence bands and the minima of the conduction bands are located at the *K* point. It should be noted that the PBE functional underestimates the band gap of 2D materials, whereas the HSE functional provides more accurate results for the band gap. However, due to its low computational cost, the PBE functional is the choice for all calculations.

**Fig. 2:**
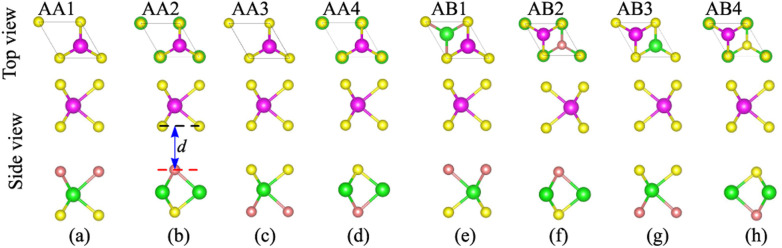
Top and side views of the atomic structures of the NbS_2_/Janus MoSSe heterostructure for different stacking patterns of (a) AA1, (b) AA2, (c) AA3, (d) AA4, (e) AB1, (f) AB2, (g) AB3 and (h) AB4.

We now build the atomic structure of the NbS_2_/Janus MoSSe heterostructure for different stacking configurations, as illustrated in Fig. 2. Owing to the small difference in the lattice parameters of NbS_2_ and Janus MoSSe monolayers, the atomic structure of the NbS_2_/Janus MoSSe heterostructure consists of a (1 × 1) unit cell of NbS_2_ and (1 × 1) unit cell of the Janus MoSSe monolayer. The lattice mismatch in the NbS_2_/Janus MoSSe heterostructure is as small as 0.6%, and it does not significantly affect the main characteristics of the heterostructure. Furthermore, due to the lack of symmetry in the two faces of the Janus MoSSe monolayer, the atomic structures of the NbS_2_/Janus MoSSe heterostructure split into two main configurations, namely NbS_2_/SMoSe and NbS_2_/SeMoS heterostructures. Each NbS_2_/SMoSe or NbS_2_/SeMoS heterostructure consists of four different stacking configurations, as presented in Fig. 2. After the geometric optimization, the interlayer distance between the NbS_2_ and Janus MoSSe layers for each stacking configuration of the NbS_2_/Janus MoSSe heterostructure is obtained, as listed in Table 1. The AB3 stacking configuration of NbS_2_/Janus MoSSe has the shortest interlayer distance, while the AA1 stacking configuration shows the largest *d*. Furthermore, to evaluate the stability of the NbS_2_/Janus MoSSe heterostructure, we calculate the binding energy (exfoliation energy)^52^ as follows:1
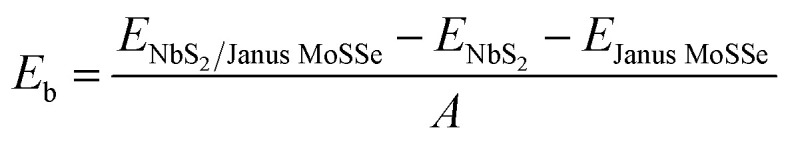
Here, *E*_NbS_2_/Janus MoSSe_, *E*_NbS_2__ and *E*_Janus MoSSe_ are the total energies of the NbS_2_/Janus MoSSe heterostructure, isolated NbS_2_ and Janus MoSSe monolayers, respectively. *A* stands for the surface area of the NbS_2_/Janus MoSSe heterostructure. The calculated binding energy values obtained from the DFT-D3 method of the NbS_2_/Janus MoSSe heterostructure for all eight stacking configurations are in the range from −32 to 20 meV Å^−2^, which are listed in Table 1. Our results show that the binding energy values are of a similar magnitude to those observed in typical van der Waals (vdW) heterostructures, including HfSeX (X = S, Se)/graphene,^53^ NbS_2_/MoSi_2_P_4_,^54^ MoS_2_/MoSSe,^35^ MoSSe/g-GeC^55^ and GaN/BP.^56^ This finding suggests that all stacking configurations of the NbS_2_/Janus MoSSe heterostructure exhibit typical vdW interactions. These weak vdW interactions are responsible for maintaining the stability of the heterostructure, which can be synthesized in experiments using various techniques, including chemical vapor deposition (CVD)^39^ and epitaxial growth.^57^ Additionally, the negative values of the binding energies of the NbS_2_/Janus MoSSe heterostructure for all stacking configurations confirm that they are energetically stable. Notably, the AB3 stacking configuration exhibits the lowest binding energy, establishing it as the most energetically stable structure. Additionally, we also used the DFT-D2 method to calculate the binding energy of the NbS_2_/MoSSe heterostructure for comparison. One can observe that the DFT-D2 binding energy is larger than that obtained by the DFT-D3 method. However, both DFT-D2 and DFT-D3 consistently predict that the AB3 stacking configuration exhibits the lowest binding energy. Furthermore, to consider the dynamic stability of the AB3 stacking configuration, we further measure its phonon spectrum, as illustrated in Fig. S1 of the ESI.† We observe that there are no negative frequencies in the phonon spectrum of the AB3 stacking configuration of the NbS_2_/Janus MoSSe heterostructure, indicating that such a configuration is dynamically stable. Hence, we focus on this stacking configuration for all the subsequent investigations.

**Table 1:** Calculated interlayer distance (*d*, Å), binding energy calculated by DFT-D3 and DFT-D2 methods (*E*_b_, meV Å^−2^), Schottky barrier heights (eV), work functions (*W*, eV) and contact types in the NbS_2_/Janus MoSSe heterostructure for the different stacking configurations

Materials	Stacking types	*d*	*E*_b_		*Φ*_n_	*Φ*_p_	*W*	Schottky type
			DFT-D3	DFT-D2				
NbS_2_/SMoSe	AA1	3.42	−21.50	−16.85	1.14	0.26	6.05	p-type ShC
	AA2	2.91	−20.33	−16.84	0.78	0.51	6.11	p-type ShC
	AB1	3.34	−31.06	−28.32	1.18	0.18	6.03	p-type ShC
	AB2	2.88	−28.15	−25.36	0.95	0.28	6.06	p-type ShC
NbS_2_/SMoSe	AA3	3.39	−21.99	−17.03	1.14	0.25	6.07	p-type ShC
	AA4	3.31	−20.74	−17.33	0.80	0.49	6.07	p-type ShC
	AB3	2.83	−32.74	−30.84	1.16	0.19	6.06	p-type ShC
	AB4	2.78	−30.16	−26.54	0.89	0.31	6.08	p-type ShC

**Fig. 3:**
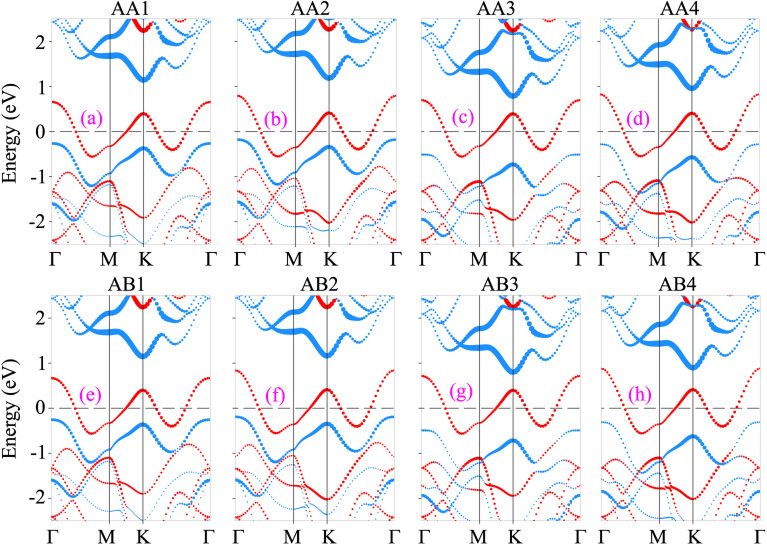
Projected band structures of the NbS_2_/Janus MoSSe heterostructure for different stacking patterns of (a) AA1, (b) AA2, (c) AA3, (d) AA4, (e) AB1, (f) AB2, (g) AB3 and (h) AB4. Red and cyan circles represent the contributions of metallic NbS_2_ and semiconductor MoSSe layers, respectively.

The projected band structures of the NbS_2_/Janus MoSSe heterostructure for all eight stacking configurations are illustrated in Fig. 3. One can find that the electronic band structures of such configurations appear to be a combination of those of the constituent NbS_2_ and Janus MoSSe monolayers. The nature of such a preservation arises from the dominance of weak van der Waals (vdW) interactions between the NbS_2_ and Janus MoSSe layers in their corresponding heterostructure. All the stacking configurations of the NbS_2_/Janus MoSSe heterostructure exhibit metallic behavior with a rough band crossing the Fermi level. By analyzing the projected band structures in Fig. 3, one can observe that a rough band crossing the Fermi level comes from the metallic NbS_2_ layer. More interestingly, upon the formation of the metal/semiconductor NbS_2_/Janus MoSSe heterostructure, either an ohmic contact (OhC) or Schottky contact (ShC) is generated, depending on the energy position of the band edges of Janus MoSSe relative to the Fermi level of the NbS_2_ layer. Hence, comprehending the characteristics of both OhC and ShC contacts plays a crucial role in enabling charge carrier injection and extraction in the metal/semiconductor NbS_2_/Janus MoSSe heterostructure. From Fig. 3, we can find that all the stacking configurations form a ShC contact because the Fermi level of the metallic NbS_2_ layer lies between two band edges of semiconductor Janus MoSSe. Based on the Schottky–Mott rule^58^ the Schottky barriers for n-type and p-type ShC contacts can be established as follows:

2 *Φ*_n_ = *E*_C_ − *E*_F_

and

3 *Φ*_p_ = *E*_F_ − *E*_V_

Here, *E*_C_, *E*_V_ and *E*_F_ are the CBM and VBM band edges of semiconductor Janus MoSSe and the Fermi level of the metallic NbS_2_ layer, respectively. The obtained Schottky barriers for all eight stacking configurations of the NbS_2_/Janus MoSSe heterostructure are listed in Table 1. One can observe that all the stacking configurations of the NbS_2_/Janus MoSSe heterostructure exhibit p-type ShC contact with the Schottky barriers ranging from 0.18 to 0.51 eV. The AB3 stacking configuration has the narrowest Schottky barrier compared to other configurations.

**Fig. 4:**
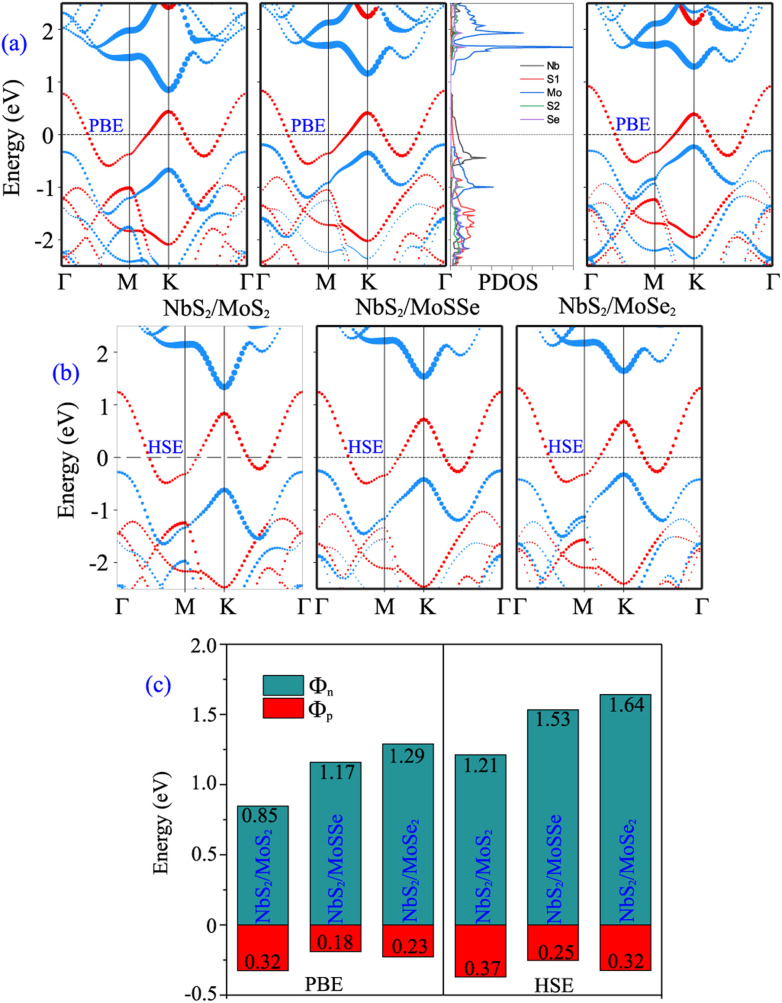
Projected band structures of NbS_2_/MoS_2_, NbS_2_/MoSSe and NbS_2_/MoSe_2_ heterostructures obtained using (a) PBE and (b) HSE functionals. (c) Schottky barriers of NbS_2_/MoS_2_, NbS_2_/MoSSe and NbS_2_/MoSe_2_ heterostructures for the most energetically stable AB3 configuration. The inset presents the partial density of states (PDOS) of all atoms in the NbS_2_/MoSSe heterostructure for the AB3 stacking configuration.

Furthermore, to determine whether the formation of the metal/semiconductor NbS_2_/Janus MoSSe heterostructure leads to a narrower Schottky barrier compared to the metal/semiconductor NbS_2_/MoS_2_ and NbS_2_/MoSe_2_ heterostructures, we next calculate the electronic properties of the latter, as depicted in Fig. 4. One can find that all the NbS_2_/MoS_2_, NbS_2_/MoSSe and NbS_2_/MoSe_2_ heterostructures form a p-type ShC contact. The *Φ*_p_ Schottky barriers for the NbS_2_/MoS_2_, NbS_2_/MoSSe and NbS_2_/MoSe_2_ heterostructures, respectively, are calculated to be 0.32/0.37, 0.18/0.25 and 0.23/0.32 eV by using the PBE/HSE functional. It is clear that the NbS_2_/Janus MoSSe heterostructure exhibits a narrower Schottky barrier compared to the NbS_2_/MoS_2_ and NbS_2_/MoSe_2_ heterostructures for both the PBE and HSE prediction. A narrower Schottky barrier results in improved device performance for those based on the metal/semiconductor NbS_2_/Janus MoSSe heterostructure. These findings illustrate that the Janus MoSSe monolayer can serve as an ideal channel when combined with the 2D metallic NbS_2_ monolayer, resulting in the formation of a metal/semiconductor NbS_2_/Janus MoSSe heterostructure that enhances charge carrier injection. Besides, for a more comprehensive understanding of the contributions of each layer in the band structure of the NbS_2_/Janus MoSSe heterostructure, we plot the partial density states (PDOS) of all atoms in the NbS_2_/Janus MoSSe heterostructure, as shown in the inset of Fig. 4(a). We can observe that the band crossing the Fermi level is mainly contributed by the Nb-orbitals of the NbS_2_ layer. Meanwhile, the Mo-orbital states contribute mainly to the CBM of the semiconducting Janus MoSSe layer and its VBM comes from the hybridization between Mo and S atoms in the MoSSe layer. In addition, to evaluate the SOC effect on the electronic properties of the NbS_2_/MoSSe heterostructure, we further plot its projected band structure with the inclusion of the SOC effect, as depicted in Fig. S2 of the ESI.† It is evident that SOC introduces a split in the valence band of the Janus MoSSe layer at the *K* point, as illustrated in Fig. S2(b).† However, it is crucial to highlight that despite the split observed in the valence band of the Janus MoSSe layer, this does not lead to a change in the contact type or barrier heights of the NbS_2_/MoSSe heterostructure because the valence band of the Janus MoSSe layer is located at the *Γ* point. Hence, the SOC effect has a negligible impact on the electronic properties of the heterostructure.

Furthermore, to investigate the charge redistribution at the interface of the NbS_2_/Janus MoSSe heterostructure, we calculate the charge density difference (CDD) as follows:

4 Δ*ρ* = *ρ*_NbS_2_/MoSSe_ − *ρ*_NbS_2__ − *ρ*_MoSSe_

**Fig. 5:**
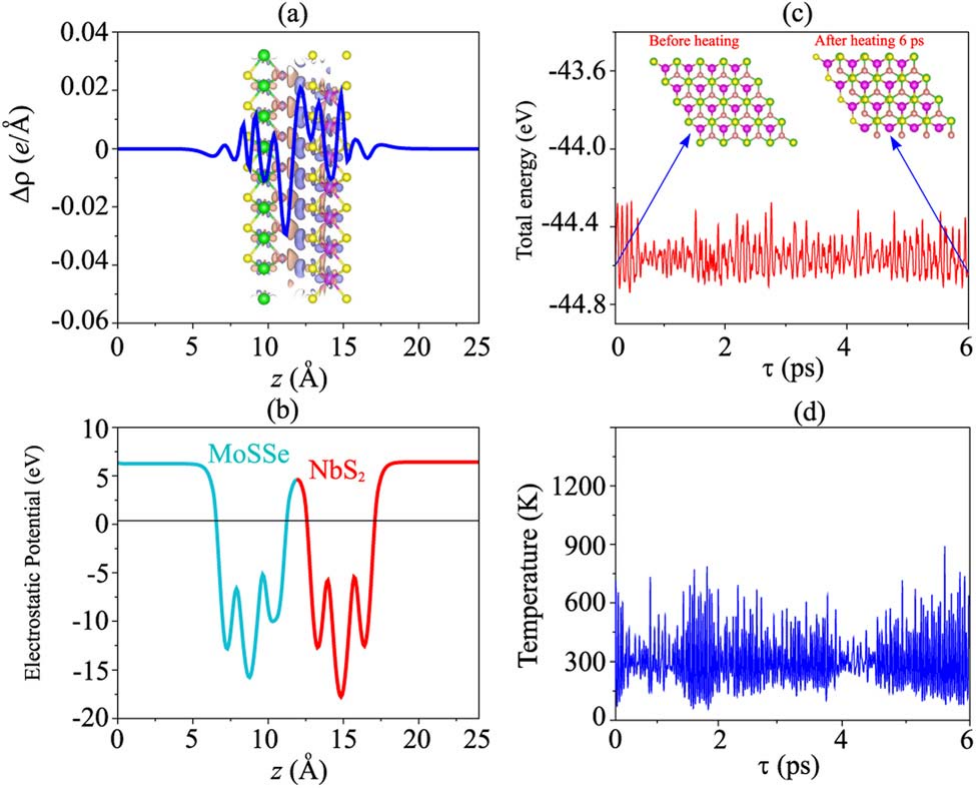
(a) Planar averaged charge density difference and (b) electrostatic potential of the NbS_2_/MoSSe heterostructure for the most energetically stable AB3 configuration. Dark purple and dark pink represent the positive and negative charges, respectively. AIMD simulations of the fluctuation of (c) total energy and (d) temperature as a function of time steps. The insets present the atomic structures of the NbS_2_/MoSSe heterostructure before and after heating for 6 ps.

Here, *ρ*_NbS_2_/MoSSe_, *ρ*_NbS_2__ and *ρ*_MoSSe_ are the charge densities of the NbS_2_/MoSSe heterostructure, isolated NbS_2_ and Janus MoSSe monolayers, respectively. The CDD at the interface of the NbS_2_/MoSSe heterostructure is depicted in Fig. 5(a). One can find that the electrons are mainly depleted on the side of the NbS_2_ layer, while they are mainly accumulated on the side of the Janus MoSSe layer. This finding demonstrates that the electrons are mainly transferred from the NbS_2_ to the Janus MoSSe layer. Such charge movement indicates that the metallic NbS_2_ layer can be used as contact electrodes for efficient electron injection, with the Janus semiconductor MoSSe layer serving as the channel in field-effect transistors (FETs) based on the NbS_2_/MoSSe heterostructure. Bader charge analysis shows that there are only 0.012 electrons, migrating from the NbS_2_ to the Janus MoSSe layer. The electrostatic potential of the NbS_2_/MoSSe heterostructure is depicted in Fig. 5(b). It is evident that the NbS_2_ layer has a higher potential than the Janus MoSSe layer in the NbS_2_/MoSSe heterostructure, confirming that the electrons migrated from the NbS_2_ to the MoSSe layer. Furthermore, the charge transfer at the interface of the heterostructure could lead to the formation of an interface dipole, which can change the barrier heights of such a heterostructure. The interface dipole can be obtained as follows:

5 Δ*V* = *W*_NbS_2__ − *W*_NbS_2_/MoSSe_

where *W*_NbS_2__ and *W*_NbS_2_/MoSSe_ are the work functions of the isolated NbS_2_ monolayer and NbS_2_/MoSSe heterostructure, respectively. From Fig. 5(b), the interface dipole at the interface of the NbS_2_/MoSSe heterostructure is obtained to be 0.08 eV. In the presence of an interface dipole, the barrier heights of the NbS_2_/MoSSe heterostructure can be rewritten as:

6 *Φ*_n_ = *E*_C_ − *W*_NbS_2__

and

7 *Φ*_p_ = *W*_NbS_2__ − *E*_V_

We observe that the barrier heights of the NbS_2_/MoSSe heterostructure remain nearly unchanged in the presence of an interface dipole compared to those without it. Such a phenomenon can be attributed to the small magnitude of the interface dipole and the minimal amount of charge transfer.

To evaluate the charge injection efficiency of the NbS_2_/MoSSe heterostructure, we further calculate its tunneling probability 
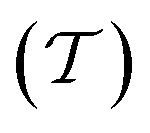
 and the tunneling-specific resistivity (*ρ*_t_) as follows:8
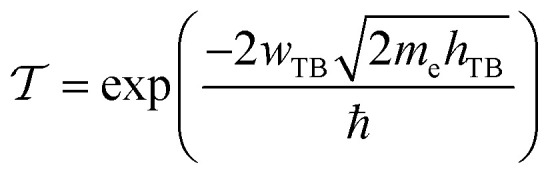
and9
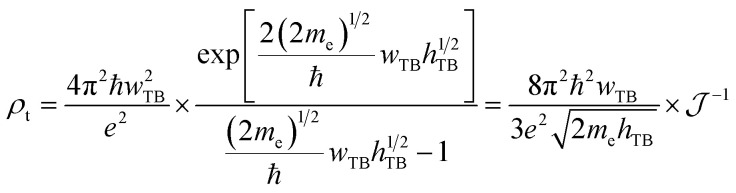
Here, *h*_TB_ and *w*_TB_ are the tunneling barrier height and width, respectively. h is the reduced Planck's constant. *e* and *m*_e_ are the electron magnitude and mass of a free electron, respectively. From the electrostatic potential of the NbS_2_/MoSSe heterostructure, the *h*_TB_ and *w*_TB_ are calculated to be 4.61 eV and 1.40 Å, respectively. Using eqn (8) and (9), the tunneling probability and tunneling-specific resistivity of the NbS_2_/MoSSe heterostructure are 3.23% and 1.89 × 10^−9^ Ω cm^2^, respectively. Interestingly, the tunneling-specific resistivity of the NbS_2_/MoSSe heterostructure is comparable to that observed in other heterostructures, including Bi/MoS_2_,^59^ Bi, Sb semimetals/TMDs,^60^ metal/MSi_2_N_4_ (M = Mo, W)^61^ and 2D (3D) metals/GeSe,^62^ suggesting that such a heterostructure could serve as an efficient contact for electronic devices.

Furthermore, to check the thermal stability of the NbS_2_/MoSSe heterostructure, we perform the *ab initio* molecular dynamics (AIMD) simulation. The fluctuations of total energy and temperature of the NbS_2_/MoSSe heterostructure for all stacking configurations as a function of time steps are displayed in Fig. 5(c, d) and S3 of the ESI.† It is evident that the fluctuations of total energy in Fig. 5(c) and S3 of the ESI† are small, and the atomic structures of the NbS_2_/MoSSe heterostructure for all stacking configurations after heating for 6 ps show no distortion or bond breaking, implying that the NbS_2_/MoSSe heterostructure is thermally stable at room temperature.

**Fig. 6:**
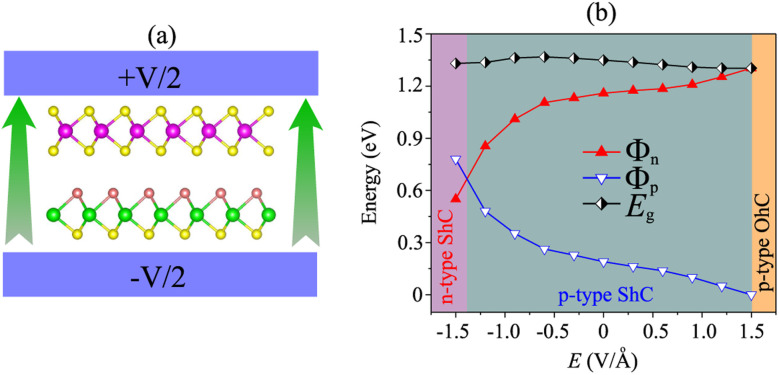
(a) The schematic model of an applied electric field along the *z* direction of the NbS_2_/MoSSe heterostructure for the most energetically stable AB3 configuration. (b) The variation of the Schottky barriers and contact types in the NbS_2_/MoSSe heterostructure as a function of electric fields.

More importantly, the ability to adjust contact characteristics in the NbS_2_/MoSSe heterostructure is crucial for its applications in next-generation electronic devices. Recently, applying electric fields is considered as an effective way to control both the electronic properties and contact characteristics of 2D heterostructures, such as graphene-based heterostructures^63–66^ and TMD-based heterostructures.^67–70^ Therefore, we further investigate the ability to adjust the electronic properties and contact characteristics in the NbS_2_/MoSSe heterostructure by applying electric fields. The schematic model of applying electric fields along the *z* direction of the NbS_2_/MoSSe heterostructure is illustrated in Fig. 6(a). The positive electric field is defined as the direction pointing from the Janus MoSSe to the NbS_2_ layers. The variation of the Schottky barriers of the NbS_2_/MoSSe heterostructure under different electric fields is depicted in Fig. 6(b). It is evident that applying an electric field varies the Schottky barriers, resulting in a transformation of the contact types in the NbS_2_/MoSSe heterostructure. The *Φ*_n_ of the NbS_2_/MoSSe heterostructure increases as the strength of the electric field increases from −1.5 to +1.5 V Å^−1^, whereas the *Φ*_p_ had decreased accordingly. This finding implies that the Schottky barriers *Φ*_n_ and *Φ*_p_ change in two opposite directions under the application of electric fields. Under the influence of a negative electric field of −1.3 V Å^−1^, we observe a reduction in the value of *Φ*_n_, causing it to become narrower than the *Φ*_p_ of the NbS_2_/MoSSe heterostructure. This change leads to a shift from p-type ShC to n-type ShC in the NbS_2_/MoSSe heterostructure. Conversely, when a positive electric field is applied, *Φ*_n_ decreases, yet it remains narrower than the *Φ*_p_ of the heterostructure, maintaining its p-type ShC characteristics. Upon further application of a positive electric field, reaching +1.5 V Å^−1^, *Φ*_n_ continuously decreases until it reaches zero, signifying a transition from p-type ShC to p-type OhC in the NbS_2_/MoSSe heterostructure.

**Fig. 7:**
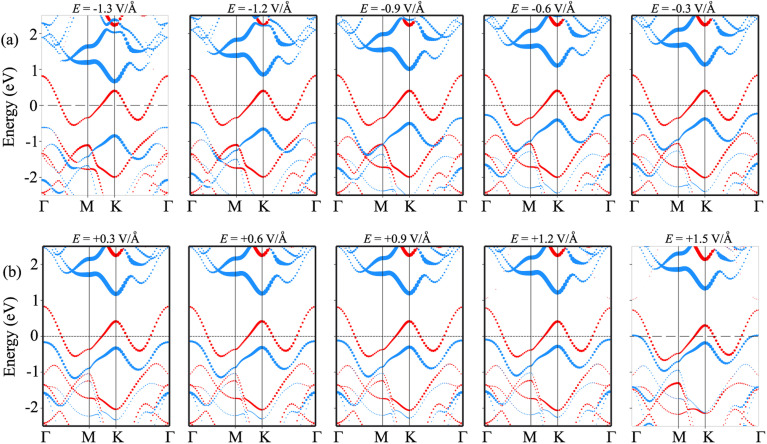
Projected band structures of the metal/semiconductor NbS_2_/MoSSe heterostructure for the most energetically stable AB3 configuration under different strengths of (a) negative and (b) positive electric fields. Red and blue lines represent the contributions of the metallic NbS_2_ and semiconducting MoSSe layers, respectively.

In order to gain deeper insight into the mechanisms underlying the change in the contact barriers and contact types in the NbS_2_/MoSSe heterostructure, we further plot its projected band structures under different strengths of both negative and positive electric fields, as illustrated in Fig. 7. The impact of a negative electric field is clearly observable on the band edges of the Janus MoSSe semiconductor. Specifically, the VBM of the Janus MoSSe semiconductor shifts to a lower binding energy, moving further away from the Fermi level. In contrast, the CBM shifts to a lower binding energy but moves closer to the Fermi level. Hence, the application of a negative electric field causes a decrease in the *Φ*_n_ and an increase in the *Φ*_p_. Under a negative electric field of −1.3 V Å^−1^, the CBM of the Janus MoSSe semiconductor is positioned closer to the Fermi level than its VBM, suggesting that the NbS_2_/MoSSe heterostructure is transformed from p-type ShC into n-type ShC. Likewise, the application of a positive electric field induces shifts in the band edges of the Janus MoSSe monolayer, although in different directions compared to a negative electric field. The VBM of the semiconducting MoSSe layer shifts closer to the Fermi level, while its CBM moves farther away from the Fermi level. Hence, under the application of a positive electric field, the VBM of the Janus MoSSe semiconductor is always positioned closer to the Fermi level than the CBM, indicating that the NbS_2_/MoSSe heterostructure maintains the p-type ShC characteristics. With a further increase in the positive electric field, the VBM of the Janus MoSSe layer continues to shift towards the Fermi level and eventually crosses the Fermi level at a positive electric field strength of +1.5 V Å^−1^. This finding demonstrates that a positive electric field results in a transformation from ShC into OhC in the NbS_2_/MoSSe heterostructure. Indeed, it's important to note that the transition from ShC to OhC in the metal/semiconductor heterostructure is a critical factor that plays a pivotal role in enhancing the performance of devices built based on the NbS_2_/MoSSe heterostructure. For instance, with the application of a positive electric field of +1.5 V Å^−1^, the tunneling-specific resistivity of the NbS_2_/Janus MoSSe heterostructure reduces to 0.82 × 10^−9^ Ω cm^2^. Hence, this transformation can have a profound impact on device characteristics and is a key consideration for optimizing device functionality and efficiency.

## 4 Conclusions

In summary, we have designed a metal/semiconductor NbS_2_/MoSSe heterostructure and systematically investigated its electronic properties and the formation of contact types using first-principles calculations. The NbS_2_/MoSSe heterostructure exhibits exceptional structural and thermal stability, making it a compelling candidate for practical applications. The NbS_2_/MoSSe heterostructure manifests p-type Schottky contacts characterized by small Schottky barriers and low tunneling resistance, signifying its substantial utility in electronic device integration. Furthermore, we found that the electronic properties, contact barriers, and contact types within this heterostructure can be controlled by manipulating the stacking configurations and applying external electric fields. Notably, negative electric fields facilitate a transition from p-type Schottky to n-type Schottky contacts, whereas positive electric fields induce a shift from Schottky to ohmic contacts. These findings offer valuable theoretical insights for exploiting the NbS_2_/MoSSe heterostructure's versatility in advancing nanoelectronic and optoelectronic device development.

## Conflicts of interest

There are no conflicts to declare.

## Supplementary Material

NA-006-D3NA00852E-s001
