# Supplementary material for: Theoretical prediction of electronic properties and contact barriers in a metal/semiconductor NbS2/Janus MoSSe van der Waals heterostructure
Source: Nanoscale Adv. 2024 Jan 9;6(4):1193–201. doi: 10.1039/d3na00852e (PMC10863720; doi:10.1039/d3na00852e)
Supplement: NA-006-D3NA00852E-s001 [file NA-006-D3NA00852E-s001.pdf]

Electronic Supplementary Material (ESI) for Nanoscale Advances.
This journal is © The Royal Society of Chemistry 2024

Supporting Information (SI)

Theoretical prediction of electronic properties and contact barriers in metal/semiconductor NbS_2/Janus MoSSe van der Waals heterostructure

P. H. Nha,[1] Chuong Q. Nguyen,[2] Nguyen N. Hieu,[3,4] Huynh V. Phuc,[5] and Cuong Q. Nguyen[3,4,*]

[1]*Faculty of Electrical Engineering, Hanoi University of Industry, Hanoi 100000, Vietnam.*

[2]*Department of Materials Science and Engineering, Le Quy Don Technical University, Hanoi 100000, Vietnam*

[3]*Institute of Research and Development, Duy Tan University, Da Nang 550000,Vietnam*

[4]*Faculty of Natural Sciences, Duy Tan University, Da Nang 550000, Vietnam*

[5]*Division of Theoretical Physics, Dong Thap University, Cao Lanh 870000, Vietnam*

*Corresponding authors: nguyenquangcuong3@duytan.edu.vn

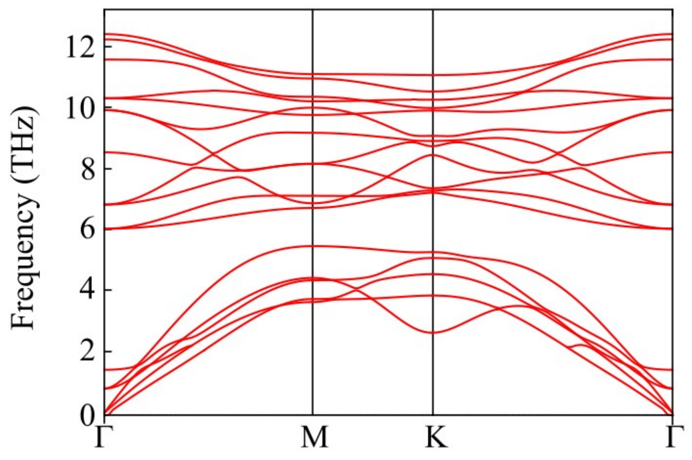

Fig. S1. Phonon dispersion curves of NbS_2/MoSSe heterostructure for the most energetically favorable stacking configuration.

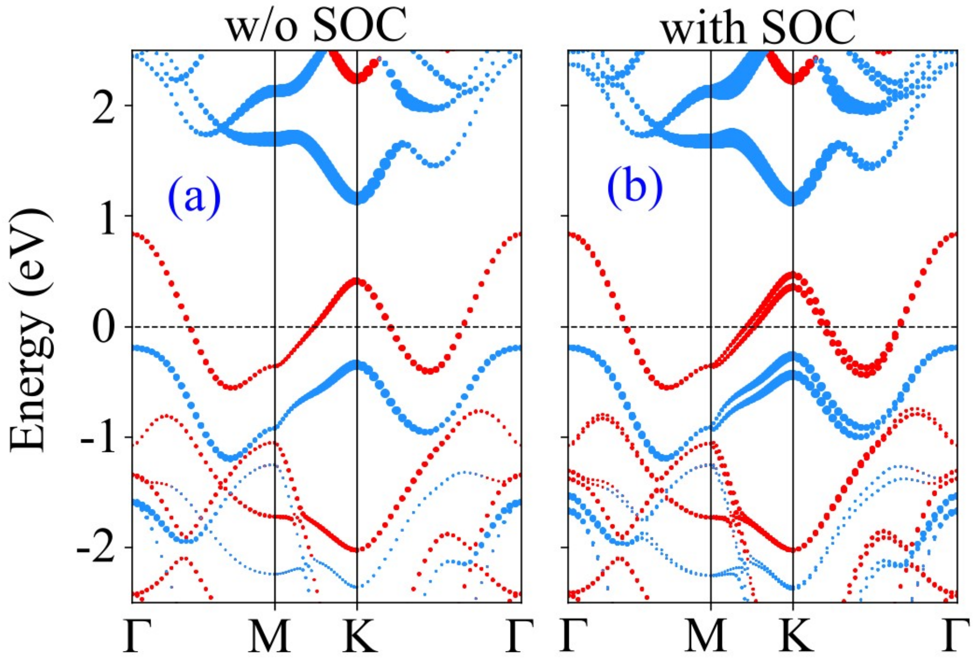

Fig. S2. Projected band structures of NbS_2/MoSSe heterostructure for the most energetically favorable stacking configuration (a) without (w/o) SOC and (b) with SOC effect.

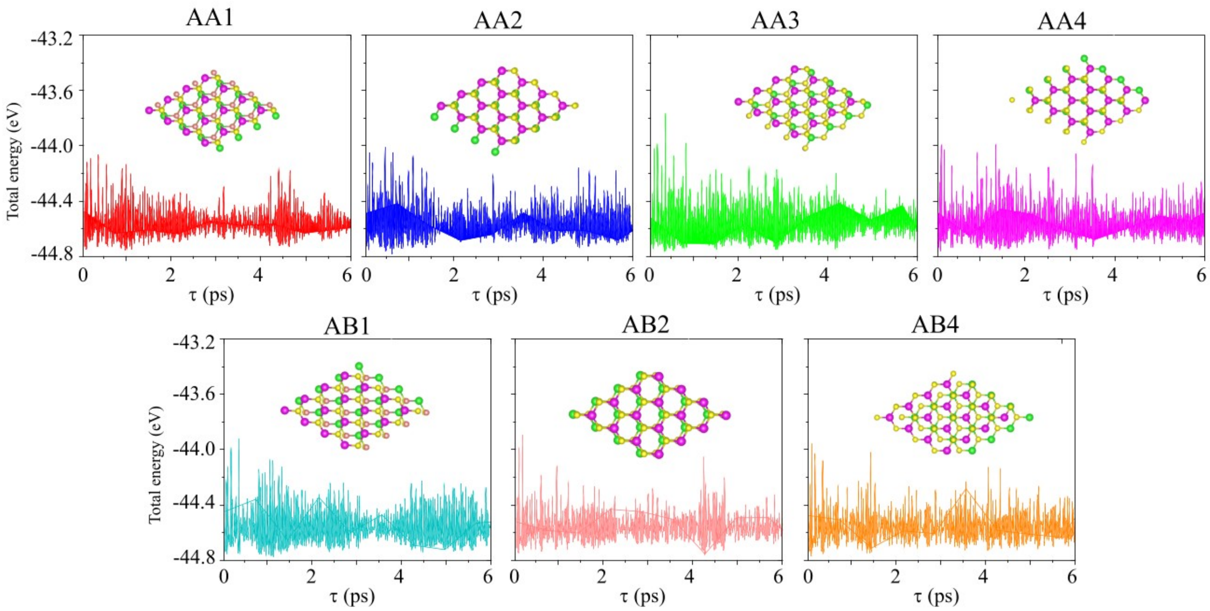

Fig. S3. The fluctuation in total energy as a function of time steps of the NbS_2/MoSSe heterostructure for different stacking configurations. The insets represent the atomic structure of heterostructure after heating to 6 ps.
